# Treatment of adenoid cystic carcinoma of the mobile tongue with anterolateral thigh flap reconstruction: A case report

**DOI:** 10.1097/MD.0000000000015250

**Published:** 2019-04-19

**Authors:** Xi Tang, Chengyao Zhang, Rui Chen, Xiaohong Zhou, Yulian Zhang

**Affiliations:** a Department of Head and Neck Cancer Center; b Chongqing Key Laboratory of Translational Research for Cancer Metastasis and Individualized Treatment; c Department of Pathology, Chongqing University Cancer Hospital & Chongqing Cancer Institute & Chongqing Cancer Hospital, Chongqing, P.R. China.

**Keywords:** adenoid cystic carcinoma, anterolateral thigh flap, functional reconstruction, tongue cancer

## Abstract

**Rationale:**

Adenoid cystic carcinoma (ACC) is an infrequent malignant neoplasm of the salivary glands. The clinical and pathological characteristics include slow growth, perineural invasion, and local recurrence. ACC of the mobile tongue is rarely reported in the literature.

**Patient concerns:**

We describe a case of a 43-year-old man patient with numbness and growth in the tongue for 1 month duration. The patient reported a long-term smoking and drinking habit.

**Diagnoses:**

A biopsy was carried out and histopathological analysis confirmed as diagnosis of adenoid cystic carcinoma. Computed tomography (CT) of the head and neck enhanced scanning revealed an ill-defined high density mass with altered enhanced signal entities involving the anterior 2/3rd of the tongue.

**Interventions:**

The patient was treated with surgery and adjuvant radiotherapy. Incision was placed over the anteriorly till 2/3rd of tongue and then the femoral anterolateral free flap was used to repair and reconstruct the defect of tongue.

**Outcome:**

The patient is currently under a postsurgical 29-month regular follow-up, showing good swallowing and speaking function without any recurrence and metastasis.

**Lessons:**

This case report suggests that surgery and adjuvant radiotherapy are suitable procedures for patients with resectable ACC of the mobile tongue.

## Abstract 2

Supplemental Digital Content is available in the text

## 1 Introduction

Adenoid cystic carcinoma (ACC) is a relatively uncommon malignant tumor that originates in the salivary glands. The peak incidence of ACC is between the fifth and seventh decades of life, which occurs predominantly among women.^[1]^ It represents approximately 1% to 2% of all malignant tumors of the head and neck and 10% to 15% of all malignant salivary gland tumors. ACC was described by Billroth as “cylindroma” and has explained its recurrent tendency in 1859. Foote and Frazell^[2]^ designated as “adenoid cystic carcinoma” in 1953. The sites of ACC include the minor or major salivary glands, and the most common minor salivary gland site is the hard palate, followed by the base of the tongue. Typical clinical performances include slow indolent growth, local recurrence, perineural invasion, and distant metastasis, mainly to the lungs and bones.^[3]^ We present a rare case of a 43-year-old male patient with a c-kit positive mobile tongue ACC treated with surgery and adjuvant radiotherapy and carried out a brief literature review on the subject.

## 2 Case report

A 43-year-old male patient presented to hospital, complaining of volume growth of the tongue with numbness for the past 1 month before consultation. The patient reported a long-term smoking and drinking habit.

This study was conducted with the approval of Medical Ethics Committee of Chongqing Cancer Hospital, and was performed in accordance with the ethical standards of the Helsinki Declaration. Written informed consents for his data and images to be used for our study and publication were obtained from patient before operation.

**Figure 1:**
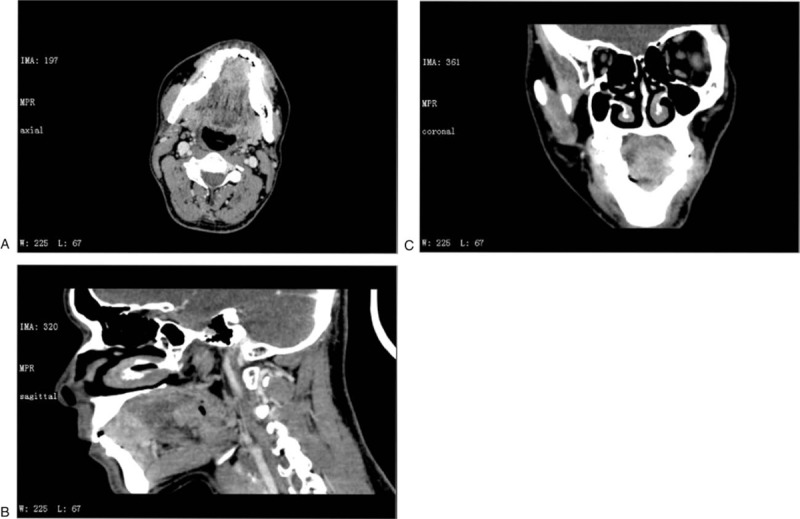
An ill-defined measuring 23 × 19 mm high density mass with altered enhanced signal entities involving the anterior 2/3rd of the tongue shown on a contrast-enhanced computed tomography. (A. axial view; B. coronal view; C. sagittal view.)

On intraoral clinical examination and palpation, an obvious smooth firm mass of about 2 cm in diameter on the mobile tongue with the same color as that of the surrounding mucosa was observed without other oral lesions. The cervical lymph nodes were not swollen on palpation. A biopsy was carried out and histopathological analysis demonstrated tissues formed by adenoid cystic carcinoma. Computed tomography (CT) of the head and neck enhanced scanning revealed an ill-defined measuring 23 mm × 19 mm high density mass with altered enhanced signal entities involving the anterior 2/3rd of the tongue. There was no obvious abnormality in the adjacent mandibular bone. No obvious enlarged lymph nodes were seen in both sides of the neck (Fig. 1). The doppler ultrasound indicated that the submental and bilateral submandibular lymph nodes were all reactive.

**Figure 2:**
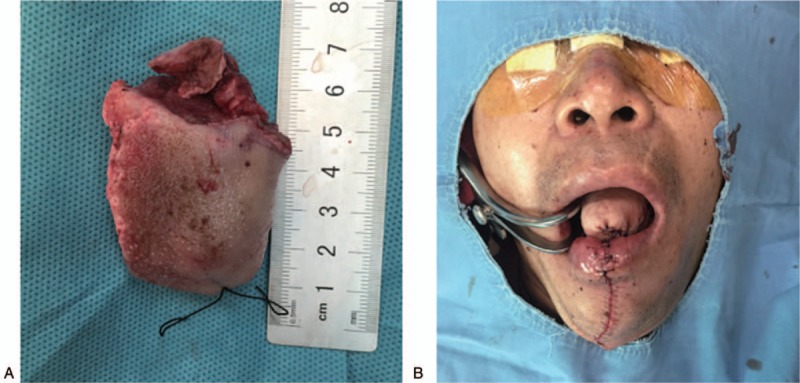
A. The resected mass is shown in an intraoperative clinical photograph. B. The tongue after reconstruction used the femoral anterolateral free flap.

The patient was positioned supine and then general anesthesia was given through nasal intubation. Incision was placed over the anteriorly till 2/3rd of tongue after emptying cervical lymph nodes at levels I–III ipsilateral to the tumor, and then the anterolateral femoral free flap (ALFT) was used to repair the defect of tongue and preserve the swallowing and speech function. During the intervention, a preventive tracheotomy was carried out to ensure breathing (Fig. 2).

**Figure 3:**
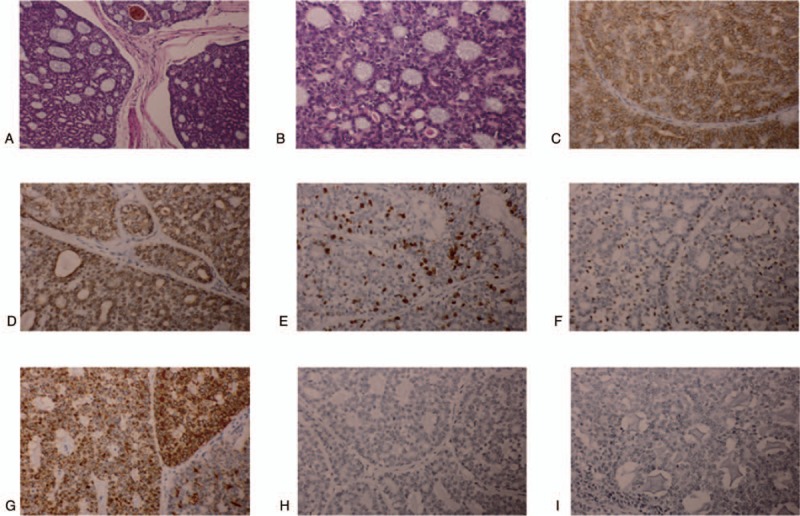
The histological analysis reveals a typical cribriform adenoid cystic carcinoma (H&E stain, A. ×100, B. ×200). C–G: Immunohistochemical features. Tumor cells are positive for CD117(C-kit), CK8, Ki-67(10%), P63, and EMA (×200). H–I: Tumor cells are negative for CEA and S-100 (×200). CEA = carcinoembryonic antigen.

A histopathological examination defined ACC with a cribriform pattern. As is typically observed in ACC, the present case was positive for CD117(C-kit), CK8, epithelial membrane antigen (EMA), Ki-67(10%), and P63, while negative for carcinoembryonic antigen (CEA) and S-100 (Fig. 3). There is only one regional lymph nodes metastasis that was found in the dissected lymph nodes located in submandibular region at levels I. Because of regional lymph nodes metastasis, postsurgical adjuvant radiotherapy was performed. We used the dose of radiation to tumor bed and lymphatic drainage area to 50 Gy.

**Figure 4:**
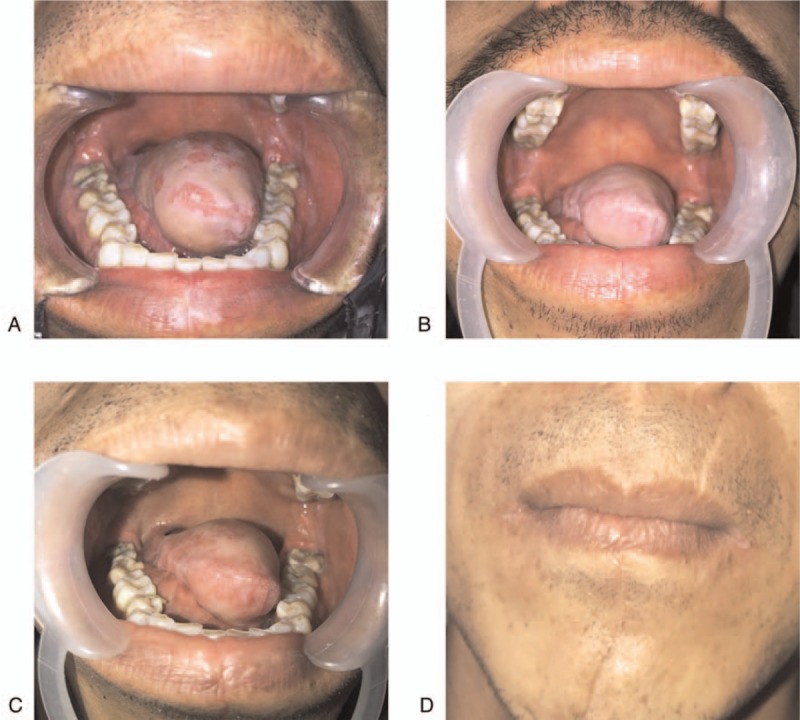
A. A photograph of the tongue after surgery 3 months. B. A photograph of the tongue after surgery 6 months. C. A photograph of the tongue after surgery 12 months. D. A photograph of the face after surgery 12 months.

The patient is currently under a postsurgical 29-month regular follow-up, showing good health without any clinically metastasis evidence. Despite the large resections these patients underwent, swallowing and speaking function were preserved at their most recent follow up appointment (Fig. 4). We also provided the video in supplement data to show the recovery of language function 6 months after surgery.

## 3 Discussion

Adenoid cystic carcinoma (ACC) has been reported to grow slowly, with a high recurrence rate, and a high chance of metastases. Perineural invasion into perineural spaces, or within the nerve itself, is a typical feature of ACC. Distant metastases often occur in the lungs and bones, and lymph node metastasis and blood dissemination are rare. Among ACCs of the head and neck regions, the site of origin has been reported to be the tongue in between 3.4% and 17.1% of cases, with the mobile tongue reported to be the site of origin in 2.9% of cases.^[4]^ The majority of ACCs are located at the base of the tongue.^[5]^ When a neoplasm is located in the tongue, the clinical manifestation for submucosal growths is typically asymptomatic. For patients with ACC of the tongue, diagnostic delay has been reported to range between 6 months and 8 years. Early diagnosis of an ACC located at the base of the tongue is difficult, and ACCs located in the mobile tongue that influence function are usually diagnosed at an earlier stage.^[5]^

Histopathologically, ACC consists of a mixture of ductal epithelial cells and myoepithelial cells, and the 2 kinds of cells are arranged in 3 different patterns, namely, solid, tubular, and cribriform. Several studies have confirmed a relationship between histologic patterns and prognosis. Huang et al^[6]^ reported 10-year survival rates of 16.7% in ACCs with a solid histological pattern and of 47.4% in ACCs with cribriform or tubular patterns. Moreover, Sequeiros et al^[7]^ reported that patients with a solid histological pattern presented with the worst prognosis, with 10-year survival rates of 34%, in contrast to 76% for the cribriform and 100% for the tubular patterns, and they suggested that the solid pattern is associated with recurrences, early distant metastases, and a higher mortality rate.

The primary treatment approach for ACC is local control, functional recovery, and distant metastasis prevention. Surgery is the mainstay of treatment, with long-term follow-up.^[1,5]^ Compared with other malignant neoplasms, ACCs are more difficult to completely remove, and positive surgical margins occur frequently.^[1]^ Tongue ACCs have rarely been reported. Luna-Ortiz et al^[5]^ found that the overall 5-year and 10-year survival rates for tongue ACCs were 51% and 34%, respectively, and that pulmonary metastases develop in 37% of patients.

Surgery for ACC of the tongue may comprise a partial glossectomy, a hemi-glossectomy, or a total glossectomy with or without reconstruction, with attention to margins and the presence of perineural invasion. Decisions concerning surgical excision should focus on patient quality-of-life outcomes, including preservation of speech and swallowing. Therefore, we suggest that large defects post-excision should be repaired with an appropriate free flap such as a radial forearm free flap or an anterolateral thigh flap (ALTF).

Reconstruction of a tongue defect is challenging, given its comprehensive functions including articulation, deglutition, and taste. Since ALTF is used in 1984, tongue reconstruction has become increasingly popular and emerged as the “workhorse flap” for tongue defect reconstruction, as this form of flap is both reliable and versatile. Other advantages of this workhorse flap are that it is easy to raise, with relatively soft tissue volume and a long vascular pedicle. The ALTF can supply a large amount of soft tissue for complex tongue defects involving the tongue and the floor of the mouth that require accurate flap design and tailoring. When selecting a flap for tongue reconstruction, an ALTF is an excellent surgical option entailing less damage at the donor site than a radial forearm flap.

Radiotherapy is routinely recommended, involving adjuvant external radiation of the surgical bed following surgery. Some studies have recommended the use of postoperative radiation for patients with advanced T stages, positive microscopic margins, or perineural invasion to decrease the incidence of local recurrence.^[8]^ In recent years, tyrosine kinase inhibitors have been used to treat ACC as an adjuvant therapeutic method in managing distant metastases. Andreadis et al^[9]^ found that most ACCs of the salivary glands stained positively for epidermal growth factor receptor, and patients with ACC might benefit from these agents, especially when surgery has failed or for those patients with recurrent or metastatic disease. Luna-Ortiz et al^[5]^ observed c-kit (CD-117) positivity in all 8 of their reported cases of ACC of the tongue.

The probability of regional lymph node metastases in ACCs of the head and neck is rare. In a retrospective study of 616 patients with ACCs located in the head and neck regions, Carrasco et al^[10]^ reported that the base of tongue, the mobile tongue, and the mouth floor were the 3 most frequent sites of lymph nodes metastasis, with incidence rates of 19.2%, 17.6%, and 15.3%, respectively, and that level Ib and II regions were the most frequently involved. The overall 5-year survival rate was significantly lower for patients with lymph node metastasis compared with those without lymph node metastasis (48% vs 77%). Selective neck dissection should be considered for patients presenting with floor-of-mouth and tongue lesions with cN0 neck and larger ACCs in these sites with lymph-vascular invasion.^[10]^ A combined method of radical surgery, including wide resection of the original tumor and radical neck dissection, followed by postoperative irradiation, is suitable for primary or recurrent cN+ patients. Factors influencing the choice of therapy are tumor site, tumor stage, histologic grade, and biologic behavior of the ACC.

The ALTF technique for tongue reconstruction has some limitations. First, restoration of the bulk, mobility, and sensibility of the tongue are significant challenges. Preservation of tongue mobility and providing tongue bulk need to be considered. To restore the swallowing function, reconstruction of tongue tissue volume is essential, and providing sufficient tissue to cover the tongue defect and restore the physiological contact tongue-palate is crucial. However, restoring the sensation function with a flap repair is difficult. If a surgeon wants to restore sensation to the tongue, an attempt may be made to connect the lateral femoral cutaneous nerve to the lingual nerve. Furthermore, excess tongue volume could reduce the oral cavity space and limit the mobility of the tongue. Recently, surgeons have reported thinning the ALTF using microscopy, in which case a skin graft is not required and tendons are not damaged. Finally, our experience has shown that when the width of a flap exceeds 9 cm, it may difficult to suture it directly to the donor site, and skin grafting is required. For defects >10 cm in width, the design of a component flap may be considered to avoid the consequences of skin grafting, due to the risk of direct suture failure at the donor site.

## Author contributions

**Conceptualization:** Chengyao Zhang, Xiaohong Zhou, Yulian Zhang.

**Data curation:** Chengyao Zhang, Yulian Zhang.

**Formal analysis:** Chengyao Zhang.

**Funding acquisition:** Chengyao Zhang.

**Investigation:** Chengyao Zhang.

**Methodology:** Xi Tang, Rui Chen, Xiaohong Zhou, Yulian Zhang.

**Project administration:** Xi Tang, Xiaohong Zhou.

**Resources:** Xi Tang, Rui Chen, Xiaohong Zhou.

**Software:** Xi Tang.

**Supervision:** Xi Tang.

**Validation:** Xi Tang, Chengyao Zhang, Rui Chen.

**Visualization:** Xi Tang, Chengyao Zhang, Rui Chen, Yulian Zhang.

**Writing – original draft:** Xi Tang, Chengyao Zhang, Yulian Zhang.

**Writing – review & editing:** Xi Tang, Chengyao Zhang.

Chengyao Zhang orcid: 0000-0001-7044-6137.

## Supplementary Material

Supplemental Digital Content
